# Obituary

**Published:** 1883-10

**Authors:** 


### OBITUARY.

Since the last issue of the JOURNAL, one of Buffalo's most honored physicians has gone to his rest. Dr. Sylvester Frederick Mixer died on Sunday, the 16th ult., at the age of sixty-eight. He was born in Morrisville, N. Y., December 27, 1815, and was graduated from the Medical Department of Yale College in 1841, and in that year began the practice of medicine in Buffalo. In 1847 he took the degree of M. D. from the College of Physicians and Surgeons of New York City. He was a member of the Medical Society of the State of New York, and of the Buffalo Medical Society, of which he was President in 1852. He was also a member of the Medical Society of the County of Erie, and of the American Medical Association; and from 1858 to 1874 was attending physician to the Buffalo General Hospital, to which institution he was subsequently appointed consulting physician.

The special meeting of the Erie County Society, which was called to take action in regard to his death, was very largely attended, and the expressions of the members present bore testimony to his high character, both as a man and a physician, and to the esteem and affection which he had honestly won from his professional brethren by a long life full of honor and integrity. The following resolutions were unanimously adopted:

It becoming, in God's providence, our afflictive duty to meet and pay honor to our departed brother, Dr. Sylvester F. Mixer, for some forty-two years a member of this society and probably its oldest member, at least resident in the city, as an expression of our sense of what is due to his memory, be it

*Resolved*, That by reason of his great worth as a man and his great excellence as a physician, we feel profoundly that we sustain by his death no ordinary loss.

2. That by reason of his marked characteristics, his acute sense of honor, delicate regard for the interests of his professional brethren, and for loyalty to his profession under all circumstances, he exemplified in this life a grand type of the blessed Christian physician.

3. That with the expression of our tenderest sympathy with the bereaved wife and family, we will regard it as a sad but sacred privilege to attend the funeral and to the last resting place the remains of our beloved brother and friend.

4. That our action upon this occasion be furnished the BUFFALO MEDICAL JOURNAL and daily papers.

---

DR. OTTO CARL BOYSEN died very suddenly September 20th, at the age of fifty-seven. He studied medicine in Keil, Germany, but owing to the political disturbances of 1848, was compelled to leave Germany before taking his degree. Upon his arrival in this country he settled at Waynesburg, Stark county, Ohio, where he practiced medicine for fifteen years. Subsequently, he went to Cincinnati, and graduated in medicine at the "College of Medicine and Surgery." In 1867, he took up his residence in Buffalo, but for some years past had declined the active practice of medicine, confining himself almost exclusively to pharmacy.

